# Waiting times in the ambulatory sector - the case of chronically Ill patients

**DOI:** 10.1186/1475-9276-12-77

**Published:** 2013-09-10

**Authors:** Leonie Sundmacher, Thomas Kopetsch

**Affiliations:** 1 Department for Economics of Primary Care, Berlin University of Technology, H80, Strasse des 17, Juni 135, 10623, Berlin; 2 Health Services Management Department, Munich School of Management, Ludwig Maximilians University, Schackstraße 4, 80539, Munich; 3 National Association of Statutory Health Insurance Physicians, Herbert-Lewin-Platz 2, 10623, Berlin

**Keywords:** Waiting times, Ambulatory sector, Consultations

## Abstract

**Aims:**

First, the influence of determinants on the waiting times of chronically ill patients in the ambulatory sector is investigated. The determinants are subdivided into four groups: (1) need, (2) socio-economic factors, (3) health system and (4) patient time pressures. Next, the influence of waiting times on the annual number of consultations is examined to assess whether the existing variation in waiting times influences the frequency of medical examinations. The waiting times of chronically ill patients are analysed since regular ambulatory care for this patient group could both improve treatment outcomes and lower costs.

**Data sources:**

Individual data from the 2010 Representative Survey conducted by the National Association of Statutory Health Insurance Physicians (KBV) together with regional data from the Federal Office of Construction and Regional Planning.

**Study design:**

This is a retrospective observational study. The dependent variables are waiting times in the ambulatory sector and the number of consultations of General Practitioners (GPs) and specialist physicians in the year 2010. The explanatory variables of interest are ‘need’ and ‘health system’ in the first model and ‘length of waiting times’ in the second. Negative binomial models with random effects are used to estimate the incidence rate ratios of increased waiting times and number of consultations. Subsequently, the models are stratified by urban and rural areas.

**Results:**

In the pooled regression the factor ‘privately insured’ shortens the waiting time for treatment by a specialist by approximately 28% (about 3 days) in comparison with members of the statutory health insurance system. The category of insurance has no influence on the number of consultations of GPs. In addition, the regression results stratified by urban and rural areas show that in urban areas the factor ‘privately insured’ reduces the waiting time for specialists by approximately 35% (about 3.3 days) while in rural areas there is no evidence of statistical influence. In neither of the models, however, does the waiting time have a documentable effect on the number of consultations in the ambulatory sector.

**Conclusions:**

In our random sample, characteristics of the health care system have an influence on the waiting time for specialists, but the waiting time has no documentable effect on the number of consultations in the ambulatory sector. In the present analysis this applies to consultations of both GPs and specialists. Nevertheless, it does not rule out the possibility that the length of waiting times might influence the treatment outcomes of certain patient populations.

## Introduction

Long waiting times for an appointment and preferential treatment for private patients are often cited by members of the statutory health insurance system as reasons for their dissatisfaction with the ambulatory sector [1]. Moreover, health service decision-makers are apprehensive that long waiting times may not only provoke discontent but also have an influence on patients’ treatment outcomes if, as a result of the longer waiting times, they consult their doctors less frequently or their illnesses are diagnosed at a later stage.

The German health insurance system is unique in the world in that it divides the population into two full insurance schemes: one statutory and one private. The statutory health insurance (SHI) is composed by autonomous health insurance organizations known as sickness funds. As of July 2013, approximately 134 non-profit statutory sickness funds are responsible for 89% of the population who are not privately insured [2]. An important element of the German SHI system is that each insured individual is entitled to the full coverage of any “necessary” treatment, under the condition that the treatment is provided economically and according to current standards of medical knowledge [3]. The remaining 11% of the population is covered by private insurance companies. This difference in category of insurance is often thought to have an influence on the waiting times since office-based physicians can charge privately insured patients for more services at higher fees.

To date, three studies have examined the influence of the category of health insurance on waiting times in the ambulatory sector in Germany. In 2006 Lüngen et al. [4] telephoned several doctors’ surgeries to make appointments either as statutory or as private health insurance patients. Statutory health insurance patients had to wait an average of three days longer for an appointment with the doctor, though the length of the wait varied considerably depending on the treatment the caller proposed. Schellhorn [5] analyzed the influence of the category of insurance on waiting times in the ambulatory sector based on the first wave of a survey now conducted regularly on behalf of the Bertelsmann Foundation’s *Gesundheitsmonitor*. Statutory health insurance system patients waited significantly longer for an appointment with a specialist. Roll et al. [6] recently used the same Bertelsmann survey for the years 2007 to 2009 to identify the influence of category of insurance, income and patient time pressures on waiting times in the ambulatory sector. They came to the conclusion that private health insurance guarantees faster access to the ambulatory sector: the privately insured waited an estimated seven days for an appointment with a specialist, compared to 16 days for the statutorily insured. For an appointment with their General Practitioners (GPs) the privately insured waited 1.4 days, while for the statutorily insured the wait was 2.3 days. Supporters of the dual insurance scheme argue that incentives to treat privately insured patients preferentially only matter if they have an influence on patients’ treatment outcomes. There are currently no secondary data available in the German health care system which combine information about waiting times with (clinically documented) information about the state of health of patients in the ambulatory sector. Consequently, there are no German studies investigating the influence of waiting times on patients’ treatment outcomes. The most active international researchers in this area are Prentice and Pizer, who in 2007 investigated the influence of waiting times on mortality on the basis of the US Veterans Health Administration (VA) data set [7]. This data set contains detailed information about ambulatory waiting times and both ambulatory and hospital treatments documented at the patient level. Prentice and Pizer showed that older veterans who experienced average waiting times of over 31 days before attending VA institutions were significantly more likely to die. In a subsequent analysis based on the same data set, Prentice and Pizer [8] examined the connection between waiting times in the ambulatory sector and hospitalizations for ambulatory care sensitive conditions. Geriatric hospital patients who attended VA institutions with an average waiting time of over 29 days were hospitalized significantly more often. Recently, Prentice et al. [9] have analyzed the effect of waiting times on the ambulatory care and health outcomes of patients with diabetes. Longer waiting times led to significantly reduced utilisation of medical facilities but had no effect on health outcomes (heart attack, rate of ambulatory care sensitive hospitalisations, etc.).

The present study pursues three objectives: First, the influence of determinants on the waiting times of chronically ill patients is investigated. The determinants are subdivided into four groups: (1) need, (2) socio-economic factors, (3) health system and (4) patient time pressures. Assuming that the variable block “need” largely approximates the state of health, the variable blocks “socio-economic factors” and “health system” should not exert systematic influence on waiting times in the ambulatory sector. Even the privately insured should not wait a significantly shorter time for an appointment with a GP or specialist since, once they have fallen ill/ are chronically ill, they should not be given priority over other patients. The standard point of reference for this is the stated aim of Social Code - Book V, to ensure care is provided consistently and in line with the needs of the insured [3]. We have limited the sample to chronically ill patients in order to investigate waiting times in a group with comparable health needs.

In the absence of data which combines information on waiting times in the ambulatory sector with patient treatment outcomes, we thus take a step back and investigate whether waiting times have an impact on the number of times that doctors are consulted by chronically ill patients within a year. There are many channels through which variation in waiting times might affect the number of consultations within a year: (1) patients with very high waiting times might wait too long to have continuous consultations throughout the year; (2) patients with long waiting times might be demotivated and skip their appointment or hesitate to make further appointments; (3) in contrast, even long waiting times do not necessarily lead to a lower number of consultations if there are well planned and organized by the practice teams. A well-managed treatment regimen with consultations on a regular basis for chronically ill patients can prevent deterioration of their condition. By the same token, however, a reduction in consultations due to longer waiting times can prove detrimental to the treatment outcome. The constriction of the sample to chronically ill patients allows investigating whether patients with need for frequent consultations receive health care on a regular basis. In the second model, with the number of consultations as a dependent variable, the hypothesis equally applies that only ‘need’ and ‘patient time pressures’ should influence the number of consultations. The annual number of visits to the doctor by chronically ill patients should not be reduced by the length of waiting times either for the statutorily or the privately insured.

Third, we investigate whether determinants of waiting times and frequency of consultations differ between urban and rural areas. Over the past few years, there has been an ongoing debate over whether the number of physicians in ambulatory care – both specialists and GPs – is sufficient to meet Germany’s present and future health care needs. Since there is currently no scientifically proven, needs-based measure to determine the optimal number of physicians, the answer to this question is controversial. Nevertheless, there is widespread agreement that certain rural areas are currently underserved or on the verge of being so. One of the major concerns is again high waiting times which might result in worse patient treatment outcomes [10]. The classification of districts as urban or rural is based on population density and the share of population in larger cities as defined by the Federal Office of Construction and Regional Planning.

## Methods

### Source of the underlying data

The data for the empirical analysis is supplied by the Representative Survey of the medically insured population carried out on behalf of the National Association of Statutory Health Insurance Physicians (KBV). Altogether 6,065 randomly selected individuals were questioned by telephone interview between May 31st and June 18th, 2010 on different aspects of ambulatory care. The number of households in the sample was proportional to the residential population in each region. The data with which the random sampling system worked included households which had no telephone book entry but could be reached by telephone on a landline number [11].

### Design of the ‘waiting times’ variable

In the present study the quantity ‘waiting times’ is the dependent variable in the first regression and the explanatory variable in the second. In the KBV Representative Survey the interviewees were asked how long they had waited between their first contact with the practice and the actual appointment in the ambulatory sector. In the German system, the principle of free choice of physicians applies to both statutory and privately insured patient who may access any GP or specialist directly or use optionally a referral form. The interviewees could quantify the length of time they had waited only in predefined intervals. Since this form of questioning severely limits the empirical analysis, we designed a continuous scale for waiting times based on these intervals. On the assumption that the waiting times cited are distributed evenly between two intervals, we used the key shown in Table 1 to translate them into a continuous variable. Following the practice of Roll et al. [6] extremely long waiting times of over four months were censored. Moreover, we ran six additional models with waiting times censored at two and three months to test the possibility that the results were driven by long waiting times of more than two or three months. The effect size of the coefficients increased slightly but sign and significance of the coefficients remained stable.

**Table 1:** Construction of the variable ‘waiting times’

**How long did it take you to get an appointment for your last visit to a doctor?**		
**Translation key**	**The lower value of the current interval + (upper value of current interval - lower value of the current interval)/2 )**	
Possible answers	Translation into days	Number of observations
Was given an immediate appointment	0	532
One day	1	58
2-3 days	2.5	108
Up to a week	5.5	119
Up to two weeks	11.0	85
Up to three weeks	18.0	49
Up to a month	26.2	42
Up to two months	44.9	32
Up to three months	74.1	34
Up to four months	102.8	13
Over four months	121.7	23
Table 1		1095

### Selection of random samples and models

The KBV Representative Survey asked interviewees about the reason for their last visit to a doctor (new symptoms, a chronic illness or preventative). Those who answered that they had last seen the doctor due to a chronic illness were included in our sample, a total of 1,095 individuals in all.

Two analyses are conducted. First, the influence of determinants on the waiting times of chronically ill patients is investigated. Next, the influence of waiting times on the annual number of visits to a doctor by chronically ill patients is analyzed. In both estimations a negative binomial model with random effects at the district level is applied since the dependent variables “length of waiting times” or “number of cases” are variables with right-skewed distribution with a variance exceeding the mean value. In view of the high percentage of interviewees who reported receiving treatment immediately, the Vuong closeness test is then performed to rule out the possibility that a zero-inflated negative binomial model would have been the better choice. Most of the explanatory variables are documented at the individual level. However, the two health system variables (doctor density, spill-over effect) are aggregated at the level of the German districts, so that the model has two levels of analysis (individual and district level). This is controlled for by means of random effects. The first estimates of the two models are carried out on the basis of the whole defined sample. To examine the different effects of the determinants in urban and rural areas, the sample is then stratified according to these two factors. The Federal Office of Construction and Regional Planning defines six basic types of regions. Of the 412 German districts, 137 are described as urban areas or areas with urban centres; 276 as rural areas. 569 of the individuals in the sample live in urban while 526 live in rural areas.

### Explanatory variables

The determinants of waiting time in the ambulatory sector are subdivided into four groups: (1) need, (2) socio-economic factors, (3) health system and (4) patient time pressures.

The ‘need’ variables ‘age’, ‘self-assessed state of health’ and ‘hospitalisation within the last 12 months’ are introduced into the regression to control for differences in individuals’ self-assessed state of health. Old age and a hospitalisation within the last 12 months are to control for more serious chronic illnesses or a generally worse state of health. It is assumed that a worse state of health reduced the waiting times. The self-assessed state of health comprises the categories ‘excellent’, ‘very good’, ‘good’, ‘moderately poor’ and ‘very poor’. Due to the low number of the observations, the last two categories, ‘moderately poor’ and ‘very poor’ are combined. Here, too, it is assumed that priority is given to treatment for the patient in a worse state of health.

Socio-economic factors are approximated in the present analysis by secondary school qualifications: the lowest (*Hauptschulabschluss*), intermediate (*Realschulabschluss*) or highest secondary school leaving certificates (*Abitur*). Theoretically, ethical considerations will prevent discrimination between patients on the basis of their socio-economic background, but in practice the level of education could have had a distorting effect when appointments were made or the illness described. Equally, the patient’s sex could have led to spontaneous prioritisation. Income was not a topic of the KBV Representative Survey.

The German health insurance system is unique in the world in that it divides the population into two insurance schemes: one statutory and one private. 89% of the German population are members of one of the non-profitmaking statutory health insurance funds because a) their income is not high enough to exempt them from compulsory membership, b) they have opted to remain in the statutory health insurance voluntarily despite exemption or c) they have been denied access to the private insurance system (with the exception of the base tariff). This last would be the case if a person exempt from compulsory statutory insurance suffered from a serious (chronic) illness and the private insurer assessed the health risk as too expensive to insure. Civil servants, students over 25 years of age and most self-employed persons are automatically exempted from compulsory statutory insurance. This difference in category of insurance could have an influence on the waiting times since office-based physicians can charge privately insured patients for more services at higher fees. A dummy variable which has a value of 1 if the person is privately insured is therefore included in the regression.

In addition to the category of health insurance, however, the structure of health care provision can also exert an influence on waiting times in the ambulatory sector. A long distance in terms of the number of minutes taken to drive to the nearest doctor can make access for patients more difficult and is therefore included in the regression. By the same token, a higher regional physician density can imply a shortening of waiting times. For districts with a spill-over effect, whereby they also provide care for a large proportion of the population in surrounding districts, increased utilisation can lead to longer waiting times in the ambulatory sector. In this regression the variable which is to model spill-over effects takes a value of 1 if the region provides care for exactly the same number of patients as live in the region. A value of 1.3, for example, indicates a region providing medical care for around 1.3 times more patients than it has residents.

Variations in waiting times are also caused by limits on individuals’ time. Wellstood et al. [12] formed focus groups to discuss the influence of individual reasons and the characteristics of the Canadian health system on access to ambulatory care. Although the focus group members referred to the problems presented by systemic barriers twice as frequently, being employed was also seen as a potential difficulty. To control for patient time pressures a dummy variable is introduced into the regression which indicates whether the person is employed (part- or full-time).

For illustration purposes the variables are grouped in logical blocks. In many cases, however, such strict separation does not reflect the reality. For example, numerous studies found higher education to be highly correlated with prevention behaviour so that the variable education most likely as well picks up behavioural characteristics. Similarly, the driving time to the nearest doctor’s surgery could (in an extreme case) be judged as a personal preference for a life remote from the care facilities of the health service. This blurring in ascription makes it generally advisable to exercise caution in interpreting the coefficients.

Last, we investigated potentially high intercorrelations between the groups of explanatory variables but did not find any evidence for the problem of multicollinearity. The pairwise correlation between “Lowest secondary school qualification” and “Private health insurance” was for instance −0.1584 and the pairwise correlation between “Intermediate secondary school” and “Private Health Insurance” - 0.1025. The pairwise correlation between “Physician density” and “Driving time” was −0.0142. None of the pairwise correlations reached a value higher than |0.2|.

### Formulation of the second model

In the second model the number of consultations within the last 12 months is regressed not only on the set of the variables defined above but also on the waiting times in the ambulatory sector, interacted additionally with the category ‘private insurance’. The interviewees were asked how many times they had consulted an office-based physician within the past 12 months. This model tests whether longer waiting times reduce the yearly number of consultations.

### Instrumentalization of the ‘waiting times’ variable

Additionally, since several studies have shown that the explanatory variable ‘waiting times’ is not exogenous to an individual’s state of health, this variable is instrumentalized. A patient’s unobserved clinical state of health influenced both individual waiting times and utilisation, as seriously ill patients are given an appointment faster and more frequently [7,8]. Although the variables defined above for the needs of a patient controlled for the state of health, they may not be able to measure this exactly enough to rule out the effect of unobserved influences. To isolate the influence of waiting times on utilisation, in this analysis the waiting time is instrumentalized with the average waiting time in the individual’s region.

## Results

Table 2 shows the descriptive statistics for the waiting times and for the number of consultations stratified by insurance category, urbanity and the interactions of the values of these two variables. Members of the statutory health insurance system wait approximately 11.6 days for an appointment with the doctor while private patients wait an average of only 5.7 days. In principle, waiting times for an appointment are shorter in urban areas (9.5 days) both for the statutorily (10.1 days) and the privately insured (5.5 days). Patients in rural areas wait an average of 12.4 days for their medical appointment. With an average waiting time of 13.1 days statutory health insurance patients in rural areas wait the longest to contact their doctor. The number of consultations is largely stable at a mean value of 12.5 visits per year. Higher values were recorded for the privately insured in rural areas (13.2 consultations per annum) and considerably lower values for the privately insured in urban areas (11.6 consultations per annum). In European comparison, only Austria and Greece have shorter average waiting times in the ambulatory sector [13].

**Table 2:** Descriptive statistics for the waiting times and number of consultations

		**Waiting times**		**Number of consultations**
	**Mean**	**Standard error**	**Mean**	**Standard error**
All	10.92	24.35	12.53	8.4
Statutory	11.56	25.09	12.75	8.31
Private	5.7	16.32	12.27	9.24
Urban population	9.52	21.81	12.84	8.52
Rural population	12.43	26.77	12.53	8.27
Statutory and urban	10.09	22.53	12.99	8.56
Statutory and rural	13.09	27.46	12.47	8.01
Private and urban	5.46	15.35	11.58	8.18
Private and rural	6.03	17.76	13.2	10.63

Table 3 shows the characteristics of the explanatory variables. At 10%, privately insured patients are slightly underrepresented in the present survey while, at 30%, those with the highest secondary school qualification are overrepresented [2,14]. In rural areas, there are significantly more individuals who report to be in poor or very poor health. At the same time there are more individuals with highest secondary school qualification and private insurance in urban areas.

**Table 3:** Summary statistics of the explanatory variables

		**All areas**	**Urban areas**	**Rural areas**
		**Percentage or mean value (standard error for mean value)**	**Percentage or mean value (standard error for mean value)**	**Percentage or mean value (standard error for mean value)**
*Need*				
Age 18 to 29		6.67	6.65	6,74
Age 30 to 44		15.89	15.46	16.34
Age 45 to 59		33.52	33.73	33.20
Age 60 to 69		20.37	22.37	18.25
Age 70 to 79		23.56	21.79	25.47
Excellent health		6.67	7.48	6.27
Very good health		13.97	15.11	12.73
Good health		47.12	45.51	48.85
Poor or very poor health		32.38	31.9	46.88
Hospitalisation within the last 12 months		34.43	34.79	34.03
*Socio-economic background* &*gender*				
Lowest secondary school qualification		33.97	33.04	34.68
Intermediate secondary school qualification		34.16	31.13	36.88
Highest	secondary school qualification	30.87	35.83	28.44
Male		41.83	42.70	40.87
Female		58.17	57.30	59,13
German citizenship		96.71	95.50	98.28
*Health system*				
Private health insurance		10.78	12.12	9.31
Most recent consultation of GP		22.37	23.19	21.48
Private health insurance * most recent consultation of GP		2.56	2,81	2.28
Driving time to nearest doctor in minutes		14	13	15
Doctors per 100, 000 inhabitants		49.59 (7.70)	50.64 (7.82)	49.36 (8.41)
Spill-over effect for neighbouring regions		1.01 (0.27)	1.00 (0.16)	0.99 (0.31)
*Patient time pressures*				
Employed		30.68	30.03	31.28
Observations		1095	569	526

Table 4 shows the results of the first negative binomial model used to estimate the influence of ‘health system’ and ‘socio-economic factors’ on the length of the waiting times with a simultaneous control for ‘need’. The coefficients indicate the incidence rate ratios which are associated with an increase in the explanatory variables by one unit. An incidence rate ratio of 1, for example, shows that the explanatory variable is a neutral factor in the distribution of waiting times between the individuals in the districts. At a value of 1.5 the increase in the explanatory variable by one unit results in the length of the waiting times for the patient increasing (relatively) by 50%. In the pooled regression the factor ‘privately insured’ reduces the waiting time for treatment by approximately 28% (about 3 days) in comparison with the statutory health insurance system. Due to the design of the interaction term, however, this only applies to waiting times for specialists. The category of insurance has no influence on consultations of a GP. Moreover, the regression results stratified by urban and rural areas show that in urban areas the factor ‘privately insured’ reduces the waiting time for specialists by some 35% (about 3.3 days) while in rural areas there is no more evidence of a significant statistical influence.

**Table 4:** Results of the negative binomial model (considering random effects) with waiting time as dependent variable

	**All areas**			**Urban areas**			**Rural areas**		
**Dependent variable: waiting time**	**IRR**	**Standard errors**		**IRR**	**Standard errors**		**IRR**	**Standard errors**	
*Need*									
Age 30 to 44	0.879	0.163		1.118	0.291		0.668	0.179	
Age 45 to 59	0.864	0.149		0.930	0.228		0.762	0.187	
Age 60 to 69	0.847	0.152		1.050	0.259		0.665	0.181	
Age 70 to 79	0.796	0.145		0.941	0.241		0.647	0.175	
Excellent health	1.285	0.254		1.356	0.362		1.371	0.411	
Good health	1.155	0.204		1.422	0.339		0.995	0.267	
Poor or very poor health	1.183	0.217		1.118	0.277		1.329	0.370	
Hospitalisation within the last 12 months	0.927	0.085		1.057	0.133		0.834	0.112	
*Socioeconomic factors* &*gender*									
Lowest secondary school qualification	0.754	0.083	**	0.663	0.098	**	0.868	0.146	
Intermediate secondary school qualification	0.849	0.088		0.724	0.103	**	1.002	0.158	
Male	0.963	0.082		0.972	0.112		0.995	0.129	
German citizenship	1.155	0.272		1.181	0.321		1.455	0.742	
*Patient time pressures*									
Employed	0.969	0.106		0.916	0.137		1.011	0.164	
*Health system*									
Privately insured	0.723	0.114	**	0.645	0.132	**	0.758	0.191	
GP consultation	0.642	0.076	***	0.514	0.085	***	0.782	0.132	
Privately insured * GP consultation	1.110	0.406		1.139	0.582		1.167	0.623	
Driving time to the nearest doctor (in minutes)	1.112	0.027	***	1.078	0.038	**	1.152	0.041	***
Number of doctors per 100 000 population	1.001	0.006		0.996	0.010		1.008	0.009	
Spill-over effect for neighbouring regions	1.005	0.161		1.000	0.361		1.000	0.180	
Constant	0.203	0.163	**	0.211	0.139	**	0.076	0.061	***
Number of observations	1095			569			526		
Number of districts	328			123			205		
Log likelihood	−2871.1877			−1469.36			−1388.6		

* Significant at the 10% level ** Significant at the 5% level *** Significant at the 1% level.

The reference individual is female, German aged 10 to 29 years, in very good health, has highest secondary school qualification, not employed and member of the statutory health insurance and last consulted a specialist.

The variable ‘driving time to the doctor’ is in all models associated positively and significantly with the length of the waiting times. The effect is an increase ranging between around 15% and 7% in the length of waiting times. Socio-economic factors have an unexpected influence on the length of waiting times: Persons with the lowest secondary school qualification have an approximately 25% shorter wait than those with the highest. A similar effect is also observable in urban areas for persons with the intermediate secondary qualification. Gender and German citizenship do not exert a systematic influence on waiting times.

Table 5 shows the results of the second negative binomial model used to estimate the influence of the length of waiting times on the number of consultations in the last year. The coefficients once again indicate incidence rate ratios. The variables for the health insurance system do not exert any systematic influence on the annual number of consultations. The variables which approximate need are significant and the patient’s own time pressures also play the expected role in the estimate of the number of consultations in the preceding year.

**Table 5:** Results of the negative binomial model (considering random effects) with number of consultations as dependent variable

	**All**			**Urban areas**			**Rural areas**		
**Dependent variable: number of consultations**	**IRR**	**Standard errors**		**IRR**	**Standard errors**		**IRR**	**Standard errors**	
*Need*									
Age 30 to 44	0.966	0.109		1.229	0.186		0.805	0.140	
Age 45 to 59	0.877	0.093		1.009	0.144		0.860	0.143	
Age 60 to 69	0.815	0.088	*	0.879	0.122		0.786	0.137	
Age 70 to 79	0.786	0.086	**	0.766	0.110	*	0.865	0.154	
Excellent health	0.997	0.130		1.174	0.212		0.778	0.150	
Good health	1.311	0.152	**	1.474	0.243	**	1.092	0.177	
Poor or very poor health	1.682	0.195	***	1.793	0.296	***	1.461	0.234	**
Hospitalisation in the last 12 months	1.344	0.061	***	1.323	0.085	***	1.398	0.096	***
*Socioeconomic factors* &*Gender*									
Lowest secondary school qualification	1.114	0.068	*	1.091	0.090		1.109	0.104	
Intermediate secondary school qualification	0.990	0.058		0.986	0.082		0.985	0.083	
Male	1.007	0.047		0.990	0.063		0.988	0.071	
German citizenship	0.921	0.148		1.079	0.207		0.608	0.186	
*Patient time pressures*									
Employed	0.785	0.046	***	0.683	0.056	***	0.869	0.074	*
*Health System*									
Privately insured	1.051	0.089		1.106	0.124		0.978	0.127	
GP consultation	0.990	0.049		1.039	0.071		0.965	0.072	
Waiting time	1.000	0.001		0.998	0.002		1.001	0.001	
Waiting time * privately insured	1.001	0.004		0.997	0.006		1.006	0.005	
Driving time to the nearest doctor (in minutes)	1.014	0.014		1.025	0.020		1.008	0.021	
Number of doctors per 100 000 population	1.000	0.000		1.005	0.005		0.995	0.005	
Spill-over effect for neighbouring regions	0.828	0.105		0.780	0.162		0.834	0.133	
Constant	5.815	3.032	**	3.101	1.371	**	14.268	7.945	***
Number of observations	540			293			247		
Number of districts	230			97			133		
Log likelihood	−1736.7257			−942.6152			−782.55849		

* Significant at the 10% level ** Significant at the 5% level *** Significant at the 1% level.

The reference individual is female, German aged 10 to 29 years, in very good health, has highest secondary school qualification, not employed and member of the statutory health insurance and last consulted a specialist.

## Discussion

First, the influence of determinants on the waiting times of chronically ill patients in the ambulatory sector was investigated. The determinants were subdivided into four groups: (1) need, (2) socio-economic factors, (3) health system and (4) patient time pressures. Second, the influence of waiting times on the annual number of consultations was then examined to assess whether the existing variance in waiting times influenced the frequency of examinations. The sample was limited to chronically ill patients since a well-managed treatment regimen with examinations on a regular basis for chronically ill patients can prevent deterioration of their condition and also lower costs. Finally, both models were stratified into urban and rural areas to test whether the determinants of interested have different effects in rural areas.

Assuming that the block of “need” variables largely approximates the state of health, in the first model the blocks of “socio-economic factors” and “health system” variables should not exert systematic influence on waiting times in the ambulatory sector. In fact, it could be shown that, beside their need, both a person’s socio-economic characteristics and their category of health insurance do indeed have an influence on the waiting time – albeit a partly unexpected one: a lower level of education reduces the waiting time. This result contrasts with European studies which have established for many countries that a high level of education facilitates access to doctors. For example, when examining waiting times for access to specialists in European countries Siciliani and Verzulli [13] found disparities related to socio-economic background. In most countries, individuals with a higher level of education waited a significantly shorter time for a consultation with a specialist than the less well-educated: the waiting time was 68% shorter in Spain, 67% in Italy and 34% in France.

Where the category of health insurance is concerned, this study has also provided evidence that the factor private health insurance shortens the waiting time. This result is in line with the results found by Schellhorn [5] and Roll et al. [6]. However, the effect by which priority was given to private patients was only significant in the case of specialists in urban areas. This may be because stronger competition among office-based doctors in urban areas leads to private patients being given higher priority and correspondingly shortened waiting times. The argument for this is that office-based doctors charge privately insured patients according to an official Medical Fee Schedule (which does not apply to statutory health insurance patients) and thus, in effect, on a fee-for-service basis. The services for statutorily insured patients, by contrast, are restricted to those in the statutory health insurance benefit catalogue, for which doctors are fully reimbursed according to a Uniform Value Scale only up to their allocated maximum ‘standard service volume’. Moreover, the official Medical Fee Schedule for privately insured patients allows the reimbursement of individual services at a considerably higher rate than can be charged on the basis of the statutory health insurance’s Uniform Value Scale and therefore raises doctors’ relative income, or at least protects it from the pressure of competition in urban areas.

Secondly, we tested whether waiting times exerted a significant influence on the number of consultations to establish whether variations in waiting times have an influence on the patients’ treatment regimen. There are numerous studies which show that continuous treatment and effective case management in the ambulatory sector can ameliorate the course of chronic disease and prevent complications [15]. Long waiting times for, or gaps in, treatment of chronic illness are thus detrimental, since a reduction of the number of consultations due to long waiting times may lower the quality of the treatment regimen and thus endanger the health of the patient. In contrast to Prentice et al. [9] who found that an increase in waiting times led to significantly reduced utilisation of medical facilities, the present study found no conclusive evidence of a significant influence exerted by waiting times on the number of physician contacts in either urban or rural areas. This result seems plausible since the average waiting times in the ambulatory sector in Germany and the estimated differences in waiting times between statutory and privately insured are rather small in European comparison [13]. However, this does not completely preclude the possibility that differences in waiting times influence the patient treatment outcome of chronically ill patients via other mechanisms than the yearly number of physician contacts. Awareness of the potential consequences of waiting times for the health of certain patient populations could usefully be exploited in the planning of appointments to improve treatment outcomes. It is therefore highly desirable that in future data be collected which combine information on waiting times in the ambulatory sector with (clinically documented) information on patients’ state of health.

The present study has carefully modelled both the influence of different factors on waiting times and the influence of waiting times on the number of consultations. However, attention should be drawn to the limitations of such modelling.

1) In the KBV Representative Survey the interviewees could state the length of their waiting times for an appointment in the ambulatory sector only in predetermined intervals. Since this form of data capture severely limits the empirical analysis, we designed a continuous scale of waiting times based on these intervals. The study by Roll et al. [6] uses similar descriptive statistics for waiting times so that the estimated information can however be considered reliable.

2) All information provided by the KBV Representative Survey was based on the interviewees’ subjective opinions. Recall bias, which applies to self-assessed health, waiting times, consultations etc., may vary with the person’s characteristics and thus have distorted results [16].

3) The analysis is based on the assumption that the reason given by interviewees for their most recent consultation of a doctor – in this case chronic illness – was correct. The information did not include any indication of the severity of the illness, which could only be approximated on the basis of the patients’ own statements concerning their state of health. Nor was any information available about the type of chronic illness, which could have been a form of diabetes requiring intensive treatment, a minor congenital heart defect, chronic obstructive pulmonary disease or, indeed, any other chronic illness.

4) Further, it was assumed that the waiting times cited for the most recent consultation were representative of other waiting times within the last 12 months. If the waiting time given for the most recent consultation was not typical, but an exceptional observation, the analysis may be correspondingly distorted.

## Competing interests

LS declares to have no competing interests. KP is employed at the Federal Physician Association and provided the data for the analysis.

## Authors’ contributions

LS planned the design and concept of the study, carried out the statistical analysis, and drafted the manuscript. KP provided the data and approved the final manuscript. Both authors read and approved the final manuscript.
